# Recurrence of *Clostridium difficile* infection in the Western Australian population

**DOI:** 10.1017/S0950268819000499

**Published:** 2019-03-18

**Authors:** M. Alfayyadh, D. A. Collins, S. Tempone, R. McCann, P. K. Armstrong, T. V. Riley, A. Cook

**Affiliations:** 1 School of Population and Global Health, The University of Western Australia, Crawley, Australia; 2 Thi-Qar Public Health Division, Ministry of Health, Iraq; 3 School of Medical and Health Sciences, Edith Cowan University, Joondalup, Australia; 4 Communicable Disease Control Directorate, Western Australian Department of Health, Grace Vaughan House, Shenton Park, Australia; 5 Department of Microbiology, PathWest Laboratory Medicine (WA), Nedlands, Australia; 6 School of Veterinary and Life Sciences, Murdoch University, Murdoch, Australia

**Keywords:** *Clostridium difficile*, recurrence, reinfection, relapse

## Abstract

*Clostridium difficile*, the most common cause of hospital-associated diarrhoea in developed countries, presents major public health challenges. The high clinical and economic burden from *C. difficile* infection (CDI) relates to the high frequency of recurrent infections caused by either the same or different strains of *C. difficile*. An interval of 8 weeks after index infection is commonly used to classify recurrent CDI episodes. We assessed strains of *C. difficile* in a sample of patients with recurrent CDI in Western Australia from October 2011 to July 2017. The performance of different intervals between initial and subsequent episodes of CDI was investigated. Of 4612 patients with CDI, 1471 (32%) were identified with recurrence. PCR ribotyping data were available for initial and recurrent episodes for 551 patients. Relapse (recurrence with same ribotype (RT) as index episode) was found in 350 (64%) patients and reinfection (recurrence with new RT) in 201 (36%) patients. Our analysis indicates that 8- and 20-week intervals failed to adequately distinguish reinfection from relapse. In addition, living in a non-metropolitan area modified the effect of age on the risk of relapse. Where molecular epidemiological data are not available, we suggest that applying an 8-week interval to define recurrent CDI requires more consideration.

## Introduction

*Clostridium difficile*, recently renamed *Clostridiodes difficile* [1], is a Gram-positive anaerobic bacillus and the most common cause of hospital-associated diarrhoea [2]. The Society of Healthcare Epidemiology of America (SHEA) considers *C. difficile* infection (CDI) as one of the most formidable infectious disease issues facing health care systems [3]. There has also been increased interest in CDI as a result of multiple outbreaks in many countries with a ‘hyper-virulent’ strain of *C. difficile* ribotype (RT) 027 [4]. Some states of the USA, the UK and Australia currently have mandated surveillance programmes for CDI [5, 6]. In Western Australia (WA), the Healthcare Infection Surveillance Western Australia (HISWA) programme commenced surveillance of hospital-identified CDI (HI-CDI) in 2010 requiring all acute care private and public hospitals to report HI-CDIs [7].

*C. difficile* has the ability to establish itself in the digestive system after the normal gut flora have been altered, such as by exposure to antimicrobials. *C. difficile* spores colonise/infect through the faecal–oral route [8]. CDI is a complex disease and, with its potential to cause ongoing symptomatic disruptions to the normal flora of the digestive system, it is often difficult to determine if patients are experiencing a relapse in infection (originating from the same strain that caused initial episode) or a reinfection (caused by a new strain or the same strain) [9–11]. Discriminating relapse from reinfection is crucial for both clinical and surveillance purposes. If infections cannot be correctly classified with a high degree of certainty, it also becomes difficult to identify risk factors associated with different strains, evaluate treatment effects and correctly quantify the true burden of CDI in the community [12].

Recommendations for determining CDI classification suggest that an interval of 8 weeks or less after the onset of a previous episode (provided that symptoms from the index episode resolve with or without therapy) indicates recurrent CDI [13–16]. If the time elapsed between two episodes of CDI is >8 weeks, then the second episode is classed as a new infection as opposed to recurrence of the original infection. These recommendations were developed as interim guidelines by an international *C. difficile* Surveillance Working Group in 2007, and variations have been adopted by many nations for surveillance purposes, including Australia [17, 18]. However, some studies have suggested that an 8-week interval does not allow sufficient discrimination of a recurrent infection as either a relapse or reinfection [5, 14, 19] and that an interval of 20 weeks might be optimal [12, 20].

Until whole genome sequencing (WGS) of *C. difficile* from clinical samples becomes routine and can provide the highest resolution data to determine strain relatedness, it is important that current recommendations and definitions are continually reviewed to ensure they have the ability to classify episodes of CDI correctly. To date, the application of the 8-week interval has not been tested for its performance in an Australian population. The objectives of this study were to: (1) use results from routinely performed ribotyping to test the accuracy of the 8-week interval; (2) evaluate different proposed cut-offs, including the 20-week cut-off; and (3) identify risk factors associated with either relapse or reinfection in the WA population.

## Materials and methods

### Setting and study population

This study was conducted in WA, one of the largest and most isolated health regions in the world. The state has an area of 2529875 km^2^ and a population of approximately 2.6 million, of which over 75% reside in the metropolitan area of Perth, the state capital (Australian Bureau of Statistics (ABS) 2017) [21]. Health services are delivered by a mix of government (public) and private healthcare providers. The majority (86%) of all acute care hospitals are government-run and clinical pathology services delivered by PathWest Laboratory Medicine (WA) (PathWest), the single public-sector pathology service provider.

### Study design

A retrospective cohort study was performed utilising routinely collected health data on CDI events from PathWest and the Communicable Disease Control Directorate, Department of Health, WA. Ethical approvals for this research were obtained from the Human Research Ethics Committees (HRECs) of the Department of Health and The University of Western Australia (approval numbers RGS0000000414 and RA/4/1/9124, respectively).

CDI cases recorded from October 2011 to July 2017 were included in the study. Patients were excluded if they were <2 years of age, had only one episode ribotyped or if they were not WA residents. Reinfections and relapses were derived from comparing the RTs causing initial and subsequent CDI episodes. Demographic and other related variables were collected, such as patient age, gender, postcode and hospital region where diagnosis occurred. CDI cases were defined as having diarrhoea with a positive PCR for *tcdB* (see below). Tests were only performed on loose or watery stool specimens, i.e. specimens that took the shape of the container into which they were placed. For the purposes of our study, we defined ‘relapse’ as ⩾2 episodes of CDI occurring ⩾1 week apart, both caused by the same RT, and ‘reinfection’ as infection with a new RT ⩾1 week after index CDI episode.

Postcodes were used to classify patients’ locations of residence into two main areas: metropolitan (Perth) *vs.* non-metropolitan using the Australian statistical geography standard remoteness structure as defined by the ABS 2017 [22]. A non-metropolitan area was defined as a remote, low population growth area, encompassing between 1% and 3% of the total Western Australian population (ABS 2007) [23].

### Detection, culture and molecular typing of *C. difficile*

CDI cases were identified as having diarrhoeal stools and a positive *tcdB* PCR result on the BD MAX™ *C.diff* platform [24]. Routine PCR ribotyping of toxigenic *C. difficile* isolates commenced in October 2011. Culture and PCR ribotyping were performed as previously described and isolates were assigned internationally recognised RTs or internal nomenclature prefixed with ‘QX’ where international number was unknown [24].

### Statistical analysis

Categorical variables were described as frequency counts and percentages, and continuous variables as medians and ranges. Univariate logistic regression analysis was initially used to investigate odds ratios (ORs) associated with outcome of relapse *vs.* reinfection. All variables were then assessed using a backward elimination procedure to assist in determining the predictive variables to be included in the final logistic regression model. All statistical testing was two-tailed and *P-*value of <0.05 was considered to be statistically significant. The *C*-statistic or the area under the receiver operator characteristic (ROC) curve (AUC) was calculated to determine optimal cut-off values. All analyses were performed in SAS version 9.4 (Copyright © 2012–2017, SAS Institute Inc., Cary, NC, USA).

## Results

**Fig. 1.**
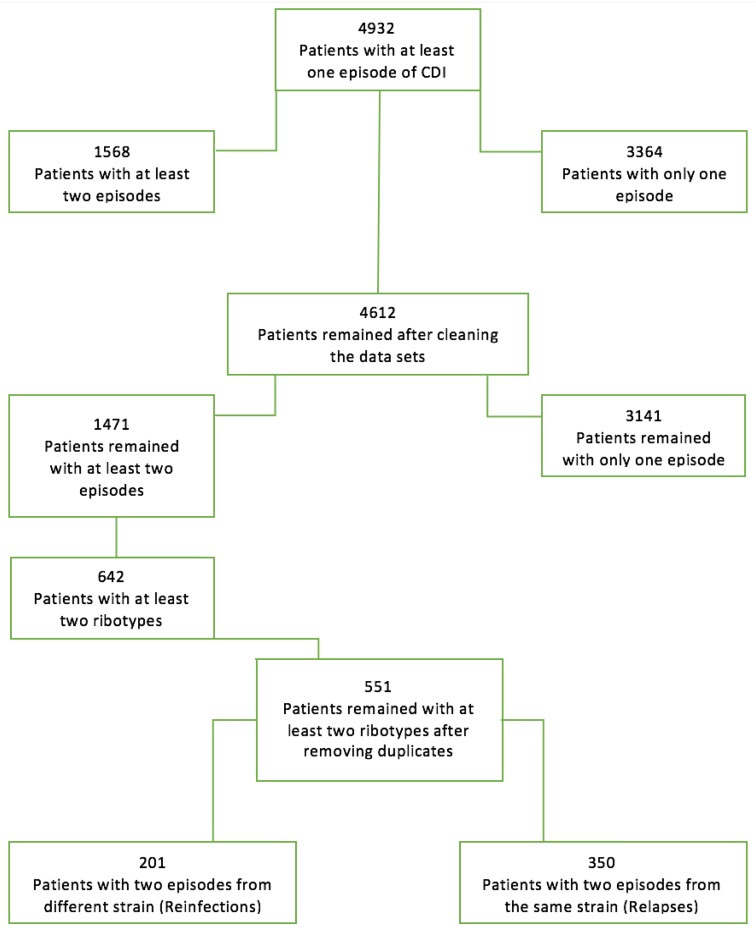
Distribution of episodes of CDI.

Of 4612 patients who were identified with CDI between October 2011 and July 2017, 1471 (32%) were diagnosed with ⩾2 episodes of CDI (Fig. 1). Overall, 1102 isolates from initial and second episodes of CDI were available from 551 patients, of which 350 (64%) were classified as relapse and 201 (36%) were classified as reinfection. The most common RTs identified are presented in Table 1, with RTs 014/020 (32%) and 002 (9%) the most dominant and significantly associated with the risk of relapse (Table 2).

**Table 1.** Distribution of RTs in 551 isolates of *C. difficile* from recurrent infections

Ribotype	Frequency	Per cent	Ribotype	Frequency	Per cent
RT 014/020	361	32.76	RT 053	12	1.09
RT 002	99	8.98	RT 081	11	1.00
RT 056	45	4.08	QX 150	10	0.91
RT 012	41	3.72	RT 001	9	0.82
RT 046	29	2.63	RT 064	8	0.73
RT 070	28	2.54	RT 251	8	0.73
RT 054	27	2.45	RT 076	8	0.73
RT 018	24	2.18	RT 072	7	0.64
RT 005	23	2.09	RT 137	7	0.64
RT 017	21	1.91	RT 247	7	0.64
RT 015/193	20	1.81	RT 087	6	0.54
QX 076	19	1.72	RT 106	6	0.54
RT 010	18	1.63	RT 078	5	0.45
RT 103	17	1.54	RT 244	5	0.45
QX 024	14	1.27	Others	194	17.60
RT 043	13	1.18

**Table 2.** Logistic regression model for risk of relapse *vs.* reinfection

Variable	Reinfection	Relapse	OR (95% CI)	*P* value	Adjusted OR (95%)*	*P* value
	(*n* = 201)	(*n* = 350)				
**Time (56 days), median (1Q, 3Q)**	**59** (**21, 190)**	**24** (**13, 69)**	**0.89** (**0.85–0.93**)	**<0**.**0001**	**0.89** (**0.85–0.94)**	**<0.0001**
**Age (20 years), median (1Q, 3Q)**	**66** (**45, 80)**	**71.5** (**54, 82)**	**1.23** (**1.06–1.43**)	**0**.**006**	**1.19** (**1.02–1.39)**	**0**.**02**
**Gender–(%)**						
** Male**	92 (46)	159 (45)	1.01 (0.71–1.43)	0.93	1.01 (0.70–1.46)	0.92
** Female**	109 (54)	191 (55)	Ref		Ref	
**Residence–(%)**						
** Non-metropolitan**	**60** (**30)**	**91** (**26)**	0.82 (0.56–1.21)	0.33	0.80 (0.50–1.29)	0.37
** Metropolitan**	**141** (**70)**	**258** (**74)**	**Ref**		**Ref**	
** Missing**	**0** (**0)**	**1** (**0.2)**				
**Location of diagnosis–(%)**						
** Metropolitan hospital**	**164** (**82)**	**292** (**83)**	**1.12** (**0.53–2.37)**	**0**.**75**	**1.07** (**0.49–2.33)**	**0**.**84**
** Non-metropolitan hospital**	**25** (**12)**	**39** (**11)**	**0.98** (**0.40–2.37)**	**0**.**97**	**1.70** (**0.45–2.99)**	**0**.**74**
** Non-hospitalised/private pathology**	**12** (**6)**	**19** (**5)**	**Ref**		**Ref**	
**8 week cut-off–(%)**						
** ⩽8 weeks**	**97** (**48)**	**236** (**67)**	2.21 (1.53–3.21)	<0.0001	—	
** >8 weeks**	**104** (**52)**	**114** (**33)**	**Ref**		—	
**20 week cut-off–(%)**						
** ⩽20 weeks**	**135** (**67)**	**300** (**86)**	2.93 (1.88–4.56)	<0.0001	—	
** >20 weeks**	**66** (**33)**	**50** (**14)**	**Ref**		—	
**Ribotype**						
** RT 014/020**	—	—	2.66 (1.77–4.01)	<0.0001	—	
** RT 002**	—	—	3.86 (1.81–8.24)	0.0005	—	

Significant findings **(*P* < 0.05)** in bold.

*Adjusted for age, gender, residence and time to second episode of CDI.

**Fig. 2.**
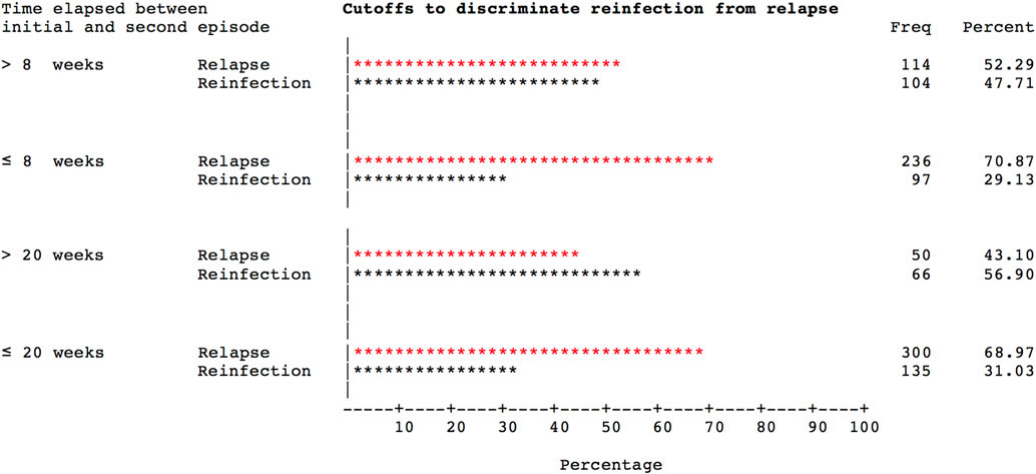
Performance of the 8- and the 20-week cut-off intervals in separating reinfections from relapses. (Reinfections) occurred within the 8- and 20-weeks intervals. Relapses occurred outside the 8- and the 20-weeks intervals.

**Fig. 3.**
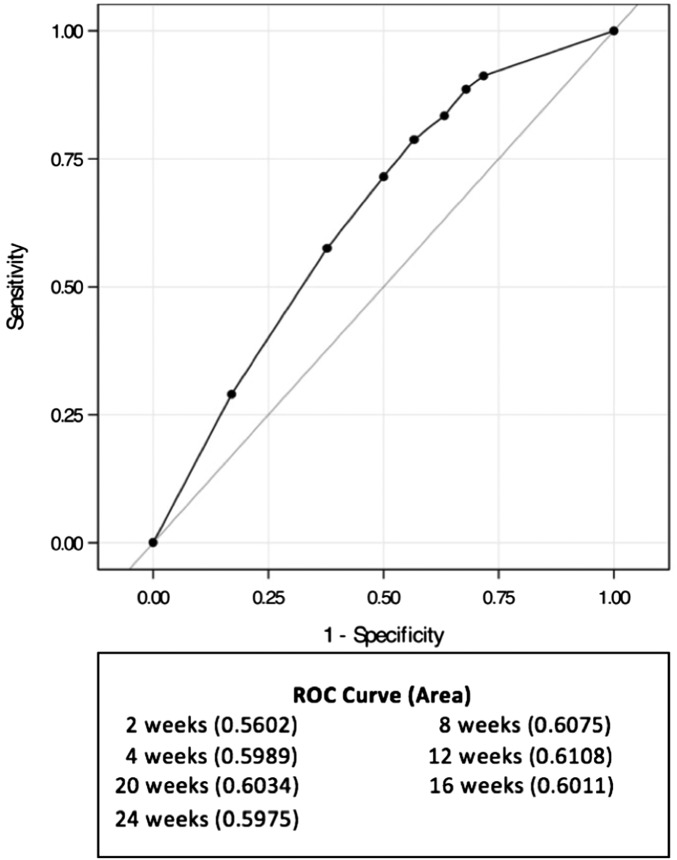
Cut-off intervals to discriminate CD reinfection from relapse. Each cut-off is used to predict reinfections. The areas under the ROC curve (AUC) indicate that the discriminatory power of all the cut-offs is not effective. The 12-week cut-off is associated with the highest AUC.

Levels of discrimination between reinfections and relapses were assessed for different cut-off intervals (Fig. 2 and Fig. 3). Within the 8-week interval, the odds of relapse (*vs.* reinfection) significantly exceeded those for infections arising beyond 8 weeks (OR 2.21, 95% CI 1.53–3.21; *P* < 0.0001). The odds of relapse (*vs.* reinfection) within the 20-week interval were also significantly greater than those for infections arising beyond 20 weeks (OR 2.93, 95% CI 1.88–4.56; *P* < 0.0001) (Table 2). Across a range of potential cut-offs, the 12-week interval was associated with the highest ROC AUC of 0.61 (Fig. 3).

Multivariate analyses indicated that age, residence (metropolitan *vs.* non-metropolitan) and time from initial diagnosis of CDI to second episode were significant risk factors for relapse. The risk the second episode being caused by a different strain increased by 11% on average for every increase of 56 days (8 weeks) following initial diagnosis of CDI to second episode (OR 1.11, 95% CI 1.06–1.17; *P* < 0.0001). There was a highly significant statistical interaction between age and residence after adjusting for time from initial diagnosis of CDI to the second episode (*P* < 0.0001) (Table 3). The risk of relapse decreased with age in years among patients from non-metropolitan areas (OR 0.98, 95% CI 0.96–0.99; *P* = 0.03) whereas patients from metropolitan areas had higher risk of relapse with age in years (OR 1.01, 95% CI 1.01–1.02; *P* < 0.0001) (Table 3).

**Table 3.** Interactions in the logistic regression model

Variable	Relapse	
	Adjusted OR (95% CI)	*P* value
Residence− × age		<0.0001
** Non-metro × age = (1 year)**	0.98 (0.96–0.99)	0.03
** Metro × age = (1 year)**	1.01 (1.01–1.02)	<0.0001
Time units (56) days	0.89 (0.85–0.93)	<0.0001

Significant findings **(*P* < 0.05)** in bold.

## Discussion

In our study population, a total of 350 (64%) individuals experienced relapse compared with 201 (36%) with reinfection. This finding is comparable with many previous studies [25–29] showing proportions of relapses between 51% and 88% compared with 12% and 41% for reinfections. These studies and the current results suggest that the proportion of relapses is greater than that for reinfections. Many early studies in distinguishing relapse from reinfection found that reinfections can be misclassified as relapses using conventional laboratory techniques [30, 31]. O'Neill *et al*. (1991), using restriction enzyme analysis of chromosomal DNA, found 75% of apparent relapses were caused by a new strain. It is currently assumed that employing a more discriminatory typing technique such as WGS would likely class some relapses as reinfections, given its ability to detect within-strain diversity [32]. The most common strain associated with relapse here, RT 014, has been divided into many sub-lineages using WGS [33].

Our findings suggest that use of various cut-off periods could result in misclassification of patients with recurrent episodes of CDI. Various studies [5, 14, 20, 25, 28, 34] support our findings that an 8-week cut-off is not effective in discriminating between reinfection and relapse. A 20-week cut-off, which was recently associated with an ROC AUC of 74% [12] implying good discriminative power, has been recommended. However, our study found a ROC AUC of only 59% for a 20-week cut-off, while a 12-week interval was associated with the highest AUC of 61%. The 12-week cut-off has been adopted by many health centres in the USA [35].

Risk factors for recurrent CDI episodes are not well understood, but can include treatment failure or continuous exposure to the same antecedents that resulted in the initial episode. Our findings of a significant difference in the risk of reinfection *vs.* relapse between younger and older patients are supported by other studies [26, 36]. We observed that the risk of reinfection tended to be higher among older patients from non-metropolitan areas, whereas it was lower among younger patients from the metropolitan area. Conversely, the risk of relapse decreased with age among patients from non-metropolitan areas and yet increased with age among those from the metropolitan area. In non-metropolitan WA, residents (such as pastoralists) are more likely to fall within older demographic groups and more commonly reside in proximity to production animals which are known reservoirs of *C. difficile* [37–42]. Risk of reinfection may also relate to changes in immunity and gut microflora as people advance in age or to differences in exposure to environmental sources. Younger patients were more likely to experience relapse in the metropolitan area, possibly because the relatively limited diversity of *C. difficile* RTs in urban environments makes reinfection with a novel strain less likely.

There were a number of strengths in our study design, including a relatively large sample size and consistency of laboratory methods over a long period using a single public sector pathology service. This study is the first in Australia to characterise relapse *vs.* reinfection using the technique of PCR ribotyping. To our knowledge, our study represents a first attempt in Australia to compare the risk of relapse *vs.* reinfection in patients from non-metropolitan areas to those from metropolitan areas.

There were some potential limitations in our study. First, we were limited by the restricted number of available risk factors included in the dataset. Other factors – such as the presence of comorbid medical conditions and the history of antibiotic use – might confound the true relationship between the risk of reinfection or relapse with time, age and residence. Second, we were unable to ascertain from the data whether the initial episode was resolved or if diarrhoeal symptoms were ongoing. ‘Test for cure’ is not performed by our laboratory and only diarrhoeal specimens are tested for CDI. We allowed for a period of ⩾1 week after initial episode to define recurrent episodes as relapse or reinfection based on comparing RTs. Clinical practice guidelines recommend a period of >14 days from initial infection to second episode to be defined as recurrent CDI [43]. However, we identified 27 cases where a new RT was isolated within 7–14 days of initial diagnosis. This is of great interest because it suggests study subjects are coming into contact with *C. difficile* on a regular basis. We have shown that *C. difficile* is increasingly prevalent in our local outdoor environment, for example, contaminating roll-out lawn [44] and retail vegetables [45] in WA. Finally, PCR ribotyping is not as powerful a tool as WGS for distinguishing discrete strains or their sub-lineages [32].

In conclusion, this study suggests that 8- and 20-week cut-offs both failed to reliably distinguish reinfection from relapse. Our results have significant implications for public health strategies aimed at controlling infection by *C. difficile*. For patients with relapse, new treatment plans may be assigned, such as faecal microbiota transplantation or courses of newer agents such as fidaxomicin [46, 47]. In comparison, reinfection might indicate some failure in applied infection prevention and control strategies in healthcare facilities, and could suggest that alternative disinfection measures might need to be considered [48]. We suggest that current definitions utilising 8- or 20-week intervals to distinguish between relapse and reinfection require reconsideration. Repeating these analyses using WGS is warranted in the future.
